# GMHAN: a heterogeneous graph attention framework for prioritizing coding and non-coding driver genes

**DOI:** 10.1093/bioinformatics/btag403

**Published:** 2026-06-18

**Authors:** Ping Meng, Tianjiao Zhang, Guohua Wang

**Affiliations:** Faculty of Computing, Harbin Institute of Technology, Harbin 150001, China; School of Computer Science and Artificial Intelligence, Northeast Forestry University, Harbin 150040, China; Faculty of Computing, Harbin Institute of Technology, Harbin 150001, China

## Abstract

**Motivation:**

Cancer, a disease of high complexity. Identifying cancer driver genes is fundamental for elucidating oncogenesis and promoting precision medicine. Currently, most approaches mainly focus on homogeneous gene networks and single-omics data, thereby mainly identifying coding driver genes while ignoring non-coding driver genes.

**Result:**

Thus, we introduced GMHAN, a novel framework based on HAN. Firstly, we integrated the three types of omics data of genes and PPI network topology feature, together with the multi-dimensional features of miRNAs. Afterwards, we used heterogeneous graph attention networks to obtain deep feature embeddings of genes and miRNAs. Finally, the deep feature embeddings are input multilayer perceptron to obtain the probability that genes and miRNAs being cancer drivers. In a comparative evaluation against seven methods, GMHAN demonstrates better performance across both pan-cancer and cancer-specific datasets, achieving higher scores in AUC and AUPR. It has confirmed its effectiveness in identifying carcinogenic drivers.

**Availability and implementation:**

The source code of GMHAN is available at: https://github.com/mping315/GMHAN and https://doi.org/10.5281/zenodo.20154736.

## 1 Introduction

The core cause of cancer lies in the accumulation of gene mutations (Vogelstein and Kinzler 1993) and poses a risk of causing human death (Sung *et al.* 2021). Mutations that directly drive cancer are called driver mutations, and the genes that carry them are called driver genes. Discovery of cancer driver genes aims to elucidate the mechanisms underlying tumorigenesis, guiding clinical treatment, and improving patient prognosis.

Currently, several publicly available databases host cancer genomic, epigenomic, and transcriptomic data, which are important to identifying and characterizing cancer driver genes. The algorithms for driver gene identification are primarily grouped into two types: mutation frequency-based and network-based methods. Among these, MutSigCV (Lawrence *et al.* 2013) employs background mutation rate correction and statistical significance testing to recognize cancer driver genes. HotNet2 (Leiserson *et al.* 2015) leverages an analysis of heat diffusion patterns for mutations in gene interaction networks to identify driver genes with significant mutations.

A lot of evidence suggests that the abnormal activation of certain cancer driver genes may be caused by epigenetic regulation, post-transcriptional modifications, and abnormal signaling pathway hierarchically (Vogelstein *et al.* 2013, Bradner *et al.* 2017, Bell and Gilan 2020, Zhao *et al.* 2022). For example, promoter hypermethylation silences tumor suppressor genes, while genome-wide hypomethylation promotes malignant cell transformation (Kulis and Esteller 2010). Recently, researchers have discovered that integrating multi-omics data may improve the ability to identify driver genes and have thus developed some algorithms (Peng *et al.* 2022b). For instance, EMOGI (Schulte-Sasse *et al.* 2021) combine pan-cancer data with PPI (Protein-Protein Interaction) network via GCN for driver gene identification. MTGCN (Peng *et al.* 2022b) introduced a multi-task learning mechanism on this basis and integrated the multi-omics features of network nodes, using Bayesian optimization to dynamically adjust task weights. MODIG (Zhao *et al.* 2022) further adopted the graph attention network (GAT) architecture, and by synchronously analyzing heterogeneous multi-omics data and five types of gene association networks, constructed a multi-dimensional driver gene identification map.

Currently, most methods aim to identify coding of driver genes. However, studies have shown that some non-coding miRNAs also exhibit certain carcinogenic effects. For instance, overexpression of miR-17–92 can synergize with MYC to accelerate tumorigenesis. miR-372 and miR-373 may inhibit the LATS2 gene, thereby counteracting the tumor-suppressive function of p53 and cooperating with RAS to induce malignant transformation in human primary cells (Garzon *et al.* 2006). This study presents a novel framework, GMHAN, which leverages gene-miRNA heterogeneous networks and graph attention mechanisms, enabling simultaneous discovery of both coding and non-coding driver genes. The framework comprises three key phases. First, we constructed a heterogeneous interaction network incorporating PPI data, miRNA-target gene regulatory relationships, and miRNA-miRNA interactions. Second, we fused gene biological features, topological characteristics from the PPI network, and miRNA functional attributes to form multi-dimensional initial feature representations. Finally, we applied heterogeneous graph attention networks to mine deep features of cancer-related genes, enabling accurate identification of both coding and non-coding driver genes.

## 2 Materials and methods

### 2.1 Overview of GMHAN

**Figure 1:**
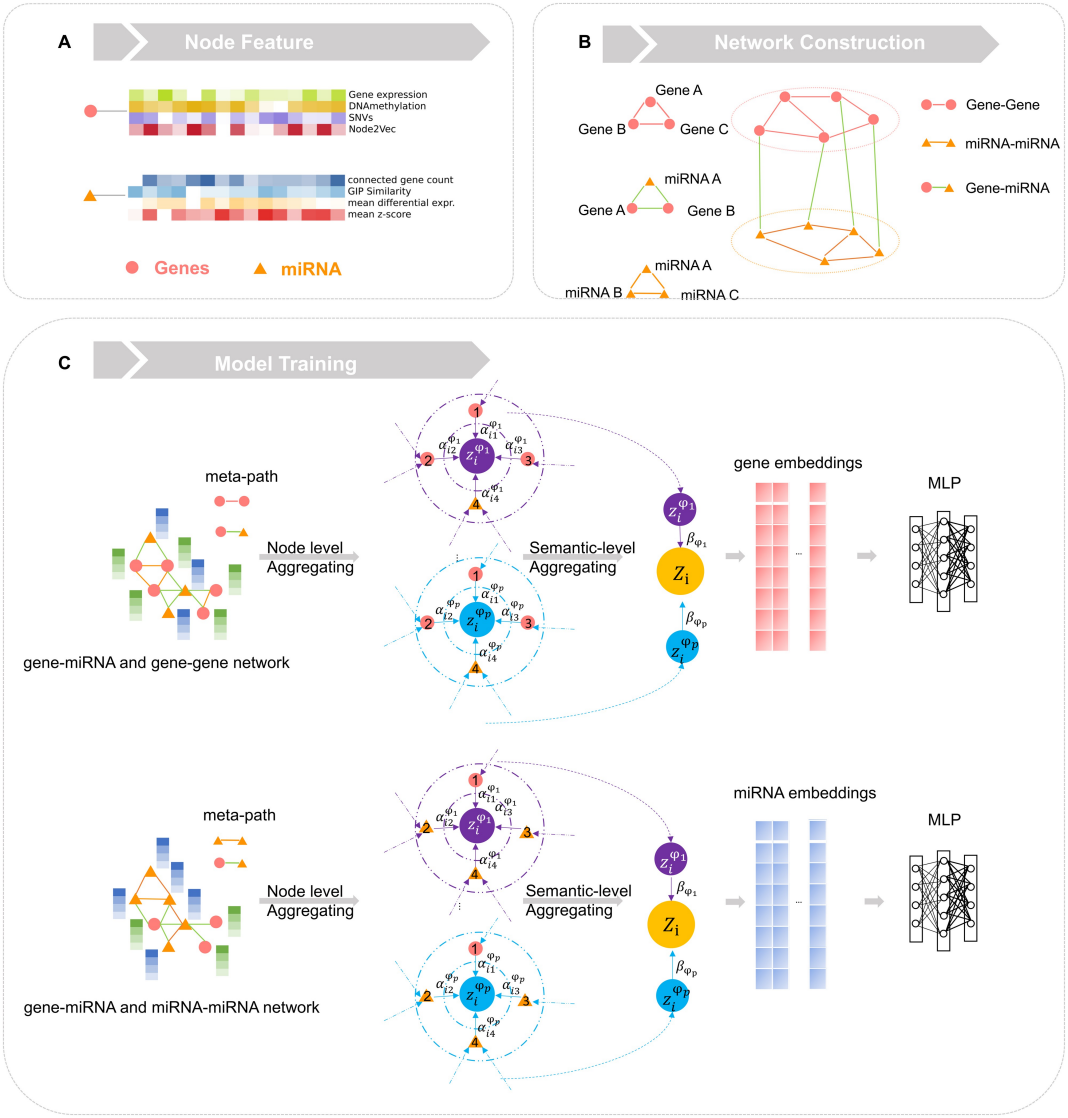
The overall process of GMHAN.

As shown in Fig. 1, the model first integrated biological features of genes, topological features from PPI network, and features of miRNAs to construct initial features for each node in the heterogeneous network. Subsequently, three distinct biological networks were constructed: (1) gene-gene network based on PPI network, (2) gene-miRNA network derived from miRNA-target interactions, and (3) miRNA-miRNA network built through functional associations between miRNAs. The feature of genes and miRNAs, along with the constructed networks, are then fed as input to the model. GMHAN employed a heterogeneous graph attention network (HAN) (Wang *et al.* 2019) architecture to predict both coding and non-coding driver genes simultaneously. Specifically, the model first learned node representations under different meta-paths, then utilized semantic-level attention mechanisms to aggregate these features into final representations. The classification task was ultimately accomplished through MLP, which output the probability predictions for all nodes.

### 2.2 Building the heterogeneous network

In this study, we leveraged the identical data resources from EMOGI (Schulte-Sasse *et al.* 2021), including the PPI network, which was sourced from the CPDB database (Kamburov *et al.* 2011). We retained edges whose scores are greater than 0.5, we ultimately obtained a network system comprising 12 944 gene nodes and 314 032 edges. Obtained multi-omics data of 16 types of cancer genes from TCGA (The Cancer Genome Atlas Program) (Weinstein *et al.* 2013), including mRNA and miRNA expression data, gene mutation, and DNA methylation data. For the miRNA network, we obtained miRNA-gene interactions from the miRTarBase (Chou *et al.* 2016), TarBase (Vlachos *et al.* 2015), and miRWalk (Dweep and Gretz 2015) databases. We retained miRNAs present in TCGA samples and genes from the PPI network, resulting in a final miRNA-gene network comprising 261 026 edges connecting 867 miRNAs with 11 451 genes. We obtained the correlation between miRNA and diseases for the HMDD v3.0 database (Huang *et al.* 2019), containing 29 685 miRNA-disease relationships involving 867 miRNAs and 853 diseases. Additionally, the miRNA-miRNA interaction network was constructed by calculating Pearson correlation coefficients of expression levels between miRNAs. Only edges with a *P* value less than 0.05 were retained. Table 1 presents the numbers of nodes and edges of the heterogeneous network.

**Table 1:** Statistical information of the heterogeneous graph.

Node	Number
Gene	12 944
miRNA	867

**Relation**	**Number**
Gene-gene	314 032
Gene-miRNA	261 026
miRNA-miRNA	294 790

The computational method of gene biological features used in this study follows the EMOGI (Schulte-Sasse *et al.* 2021). For specific calculation methods, please see the Supplementary Material. Consequently, each gene obtains a 48-dimensional biological feature vector composed of 16-dimensional gene mutation frequency, 16-dimensional methylation difference rate, and 16-dimensional gene differential expression value. We performed the node2vec algorithm on the PPI network to obtain 16-dimensional topological structure features. These topological features were then concatenated with 48-dimensional biological features, and each gene has a 64-dimensional feature vector. The miRNA feature extraction adopted is consistent with that used in GM-GCN (Peng *et al.* 2023). The features of miRNAs comprised the following components: the mean z-score computed from miRNA expression levels, the arithmetic mean of miRNA expression values, and a similarity matrix computed using the Gaussian Interaction Profile (GIP) kernel function from miRNA-disease association (Cheng *et al.* 2021, Peng *et al.* 2022, 2022a), formulated as:


(1) $$\begin{array}{c} \text{MS}(m_{i},m_{j})=\text{exp}\;\left(-\gamma_{m}\|{\text{IP}}_{m_{i}}-{\text{IP}}_{m_{j}}{\|}^{2}\right),\\ \gamma_{m}=\gamma_{m}^{\prime }/\left(\frac{1}{n}\sum_{i=1}^{t}\|{\text{IP}}_{m_{i}}{\|}^{2}\right) \end{array}$$

Through linear transformation of the Gaussian kernel similarity matrix, we obtained a 16-dimensional feature representation. This is then concatenated with the number of target genes per miRNA, resulting in a final 49-dimensional feature vector. Following this, we performed min-max normalization on the feature matrix.

In this study, 786 driver genes from the NCG 6.0 database (Repana *et al.* 2019) were utilized as the positive sample set. Similarly, cancer type-specific driver genes are from the NCG database. Negative samples were obtained by recursively eliminating genes from the complete gene set that met any of the following criteria: (1) genes annotated in the NCG database; (2) genes involved in ‘pathways in cancer’ from the KEGG database; (3) genes documented in the OMIM database; (4) genes identified by MutSigdb analysis; and (5) genes demonstrating cancer-related expression patterns. This rigorous filtering process yielded 1989 high-confidence negative samples. For non-coding RNAs, miRNA-tumor development associations were determined based on the OncomiR database (Wong *et al.* 2018). A total of 310 miRNAs with documented tumor development relevance were designated as positive samples, while 557 miRNAs without such evidence were classified as negative samples. The driver miRNAs for each cancer type were also obtained from the OncomiR database.

### 2.3 Learning node features from the heterogeneous network

#### 2.3.1 Meta-path selection

The heterogeneous graph has multiple node and edge types. Formally, let graph G = (V, E, A, R), and $V=\{V^{g},V^{m}\}$ represents nodes, $V^{g}$ denotes genes, $V^{m}$ denotes miRNAs, and $E=\{E^{\left(g-g\right)},E^{\left(g-m\right)},E^{\left(m-g\right)},E^{\left(m-m\right)}\}$ represents edges, consist of gene-gene interactions $E^{\left(g-g\right)}$, gene-miRNA interactions $E^{\left(g-m\right)}$, miRNA-gene interactions $E^{\left(m-g\right)}$, and miRNA-miRNA interactions $E^{\left(m-m\right)}$. A represents the node type mapping function ϕ(v):V→A and R represents the edge type mapping function ϕ(e):E→R, satisfying the condition |A|+|R|>2. The meta-path φ is denoted as $A_{1}\overset{R_{1}}{\to }A_{2}\overset{R_{2}}{\to }\ldots \overset{R_{l}}{\to }A_{l+1}$. It defines the combination relationship $R=R_{1}{}^{\circ}R_{2}{}^{\circ}\ldots R_{l}$, where the symbol ° represents the composition operation. The meta-paths used in this study and their interpretations are presented in Table 2. G-G and G-M-G meta-paths mainly capture semantic associations among genes, while M-M and M-G-M meta-paths mainly capture semantic associations among miRNAs. These meta-paths cover the direct interactions and indirect regulatory relationships between genes and miRNAs. Taking the G-M-G meta-path as an example, when two genes are connected through a specific miRNA, a meta-path Gene i—miRNA—Gene j is formed. In the process of message propagation, the information transmitted to Gene j incorporates both Gene i feature and the semantic information from the intermediate miRNA.

**Table 2:** Meta-paths used in our study.

Meta-path	Node sequence	Biological meaning
G-G	Gene-Gene	the direct interaction between genes
G-M-G	Gene-miRNA-Gene	the indirect association of genes mediated by miRNAs
M-M	miRNA-miRNA	direct miRNA co-regulation and functional similarity
M-G-M	miRNA-Gene-miRNA	the indirect functional association of miRNAs formed by targeting the same gene

#### 2.3.2 Node-layer attention

Given that different types of nodes possess distinct feature dimensions, projecting all nodes features of heterogeneous nodes into a unified feature space through linear transformation. In this study, we used linear transformation on the features of miRNAs, mapping their dimensionality to 64, and the formulation is:


(2) $$h_{i}^{\prime }=M_{i}\cdot h_{i}$$

Where $h_{i}$ represents the original vector, $h_{i}^{\prime }$ represents the transformed vector, and $M_{i}$ represents the transformation matrix.

Through node-level attention, we obtained the importance of each node and assigned it different weights. For the node pairs (i, j) linked through the meta-path φ, the attention score is used to represent the correlation between node j and node i, and the formulation is:


(3) $$e_{ij}^{\varphi }={\text{att}}_{\text{node}}(h_{i}^{\prime },h_{j}^{\prime };\varphi )=\sigma (a_{\varphi }^{T}\cdot [h_{i}^{\prime }\|h_{j}^{\prime }])$$

Then, we normalized the attention score of node j using the softmax function, and the formulation is:


(4) $$\alpha_{ij}^{\varphi }=\mathrm{softmax}(e_{ij}^{\varphi })=\frac{\;\text{exp}\;\left(\sigma (a_{\varphi }^{T}\cdot [h_{i}^{\prime }\|h_{j}^{\prime }])\right)}{\sum_{k\in N_{i}^{\varphi }}\;\text{exp}\;\left(\sigma (a_{\varphi }^{T}\cdot [h_{i}^{\prime }\|h_{k}^{\prime }])\right)}$$

Where $N_{i}^{\varphi }$ denotes neighbor nodes along the meta-path φ , σ represents the activation function.

To obtain the embedding for node i in the meta-path φ, we first compute attention coefficients between node pairs. These learned coefficients are then used to weight and aggregate the features of neighbor nodes, and the formulation is:


(5) $$Z_{i}^{\varphi }=\mathrm{ELU}\left(\sum_{j\in N_{i}^{\varphi }}\alpha_{ij}^{\varphi }\cdot h_{j}^{\prime }\right)$$

Then extended the above formula into the multi-head attention mechanism. That is to say, iterate the node-level attention process K times, and finally concatenate the vectors horizontally to obtain the final vector representation.

#### 2.3.3 Semantic-level attention

After obtaining node embeddings derived from individual meta-paths, we employed a global fusion mechanism based on semantic attention to integrate the node representations from multiple meta-paths. This approach first applied nonlinear transformations to the semantic-specific node embeddings to capture the importance of each meta-path. The importance of each semantic-specific embedding is then measured by calculating its similarity using a semantic-level attention, followed by normalization through the softmax function. Finally, the fused node representation is generated through the weighted summation of all meta-path embeddings.


(6) $$W_{\varphi_{i}}=\frac{1}{\left|V\right|}\sum_{v\in V}q^{T}\cdot \tanh(W\cdot Z_{v}^{\varphi_{i}}+b)$$

(7) $$\beta_{\varphi_{i}}=\frac{\;\text{exp}(W_{\varphi_{i}})}{\sum_{j=1}^{P}\;\text{exp}\;(W_{\varphi_{j}})}$$

(8) $$Z=\sum_{i=1}^{P}\beta_{\varphi_{i}}\cdot Z_{\varphi_{i}}$$

Where *W* and *b* represent the weight matrix and the bias vector, and *q* is the semantic-level attention vector, *V* is nodes count.

### 2.4 Model training

Ultimately, we implemented the node classification task using a two-layer MLP. We employed the cross-entropy loss function to calculate the loss value. For gene node classification, the formulation is:


(9) $$L_{g}=-\sum_{c=1}^{2}y_{g,c}\;\text{log}({\hat{y}}_{g,c})$$

For miRNA node classification, the formulation is:


(10) $$L_{m}=-\sum_{c=1}^{2}y_{m,c}\;\text{log}({\hat{y}}_{m,c})$$

The total loss function is:


(11) $$L_{\text{total}}=L_{g}+L_{m}$$

Where $y_{g,c}$ and $y_{m,c}$ are the true label of gene and miRNA. For the true class, its value is 1, and for all other classes, its value is 0. ${\hat{y}}_{g,c}$ and ${\hat{y}}_{m,c}$ are the predicted probability by the model that the sample belongs to the *c*-th class. We implemented the GMHAN model based on Python 3.9 and PyTorch 2.3.1 framework. The model adopts HAN architecture with the hidden layer dimension set to 256. We trained the model employing the Adam optimizer for up to 1000 epochs. The key hyperparameters were set as follows: a learning rate of $3\times 10^{-3}$, a weight decay of $5\times 10^{-4}$, and a dropout rate of 0.2.

We used 5-fold CV to rigorously assess the model’s predictive capabilities. We randomly split the datasets into five folds. In each round of validation, we reserved one subset for testing and utilized the other four for training. This design guarantees that every data point was included in a test set precisely one time. By aggregating the outcomes from these five independent runs, we obtained a stable performance average. This method effectively controls for the bias inherent in a single random train-test split, giving us greater confidence in the findings.

## 3 Results

### 3.1 Accuracy analysis of pan-cancer driver gene identification

To quantify predictive performance, we evaluated our model using AUC and AUPR. For comparison, we selected seven baseline models which designed for both homogeneous and heterogeneous networks. These seven methods include classical models (Mashup+SVM, GCN, GAT, HGT) and driver gene identification methods (EMOGI, MTGCN, MCDHGN) that have been developed. All methods were constructed with the same network structures and features. We configured their parameters using either recommended defaults or values obtained through our own optimization procedures. For detailed method descriptions and parameters, please refer to the Supplementary Material.

**Figure 2:**
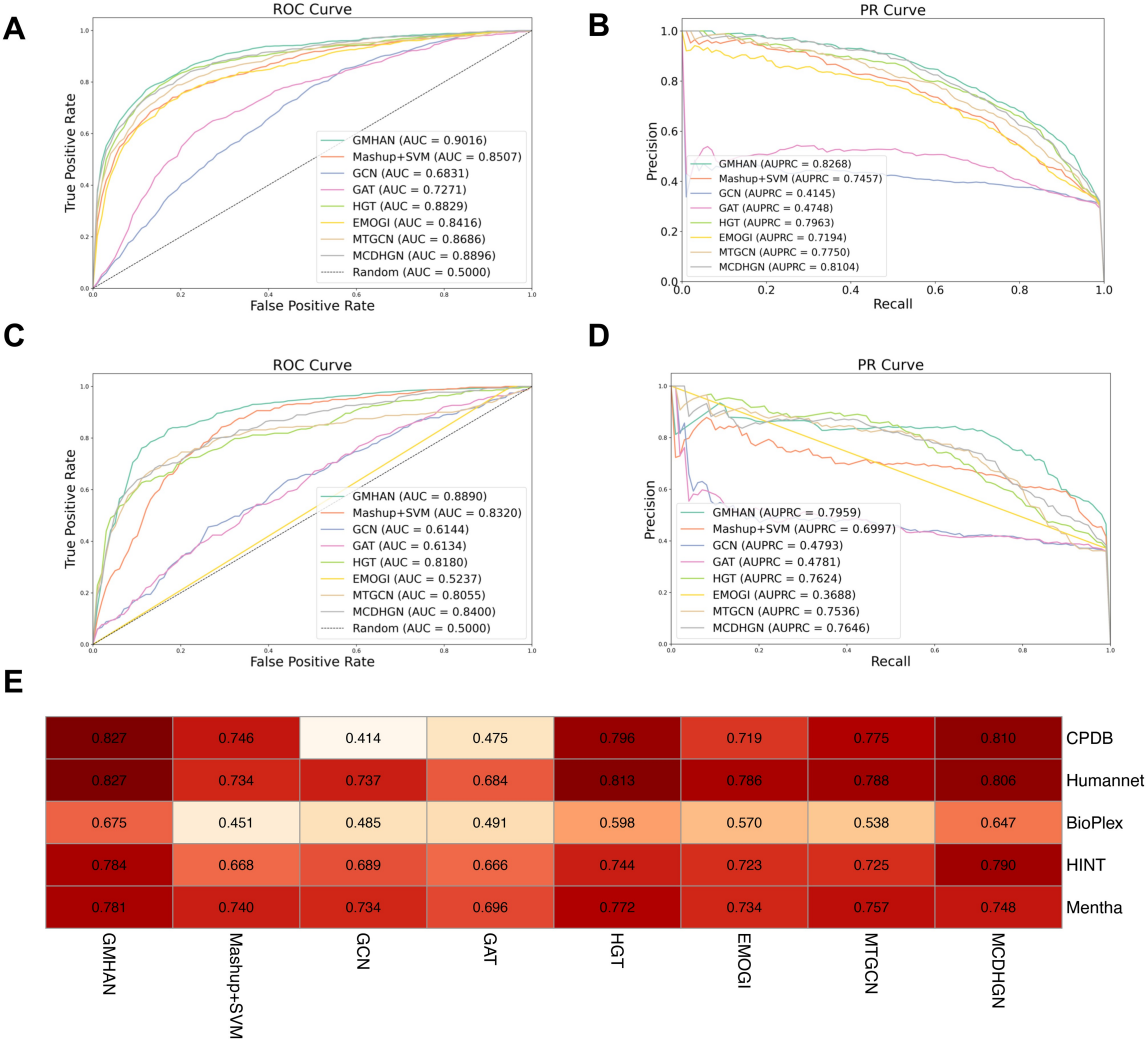
Performance comparison between GMHAN and other methods across multiple networks. (A) ROC curves for gene data. (B) PR curves for gene data. (C) ROC curves for miRNA data. (D) PR curves for miRNA data. (E) Comparative heatmap of AUPR values for various methods across different PPI networks, where color intensity is positively correlated with AUPR values (darker shades indicate higher performance, while lighter shades represent lower performance.

We trained the GMHAN model and obtained the results of both genes and miRNAs simultaneously. Results showed that GMHAN outperforms other baseline methods in classifying both types of driver genes. Specifically, for coding driver genes, the AUC value of GMHAN is 0.902 (Fig. 2A) and its AUPR value is 0.827 (Fig. 2B), leading MCDHGN by 0.012 and 0.017. For non-coding driver genes, GMHAN attained an AUC of 0.889 (Fig. 2C) and an AUPR of 0.796 (Fig. 2D), leading MCDHGN by 0.049 and 0.031. In this study, the method developed based on driver gene identification has better AUC and AUPR values than the classical models when identifying coding driver genes and non-coding driver genes. This might be because GMHAN can better learn the interactions between features and thus capture more complex information. Furthermore, we observed that the results of the heterogeneous network models are superior to the homogeneous network models. The reason might be that heterogeneous networks incorporate richer semantic information. The difference between GMHAN and other heterogeneous network models is that GMHAN can predict both coding and non-coding driver genes simultaneously, while other methods require separate processing.

To assess the model’s robustness, we trained the GMHAN on multiple PPI networks derived from diverse sources and compared it against several existing methods. As shown in Fig. 2E, the vertical axis lists GMHAN and seven baseline methods, the horizontal axis shows five different PPI networks, and the color intensity represents the AUPR for predicting coding driver genes. GMHAN achieves the highest AUPR on CPDB, HumanNet, BioPlex, and Mentha, with an average improvement of 0.017 over the best-performing baseline. On HINT, GMHAN ranks second. In addition, GMHAN performs well and stably across PPI networks of different sizes, indicating that its performance does not rely on a specific network structure. These results suggest that GMHAN is robust across heterogeneous PPI data.

**Figure 3:**
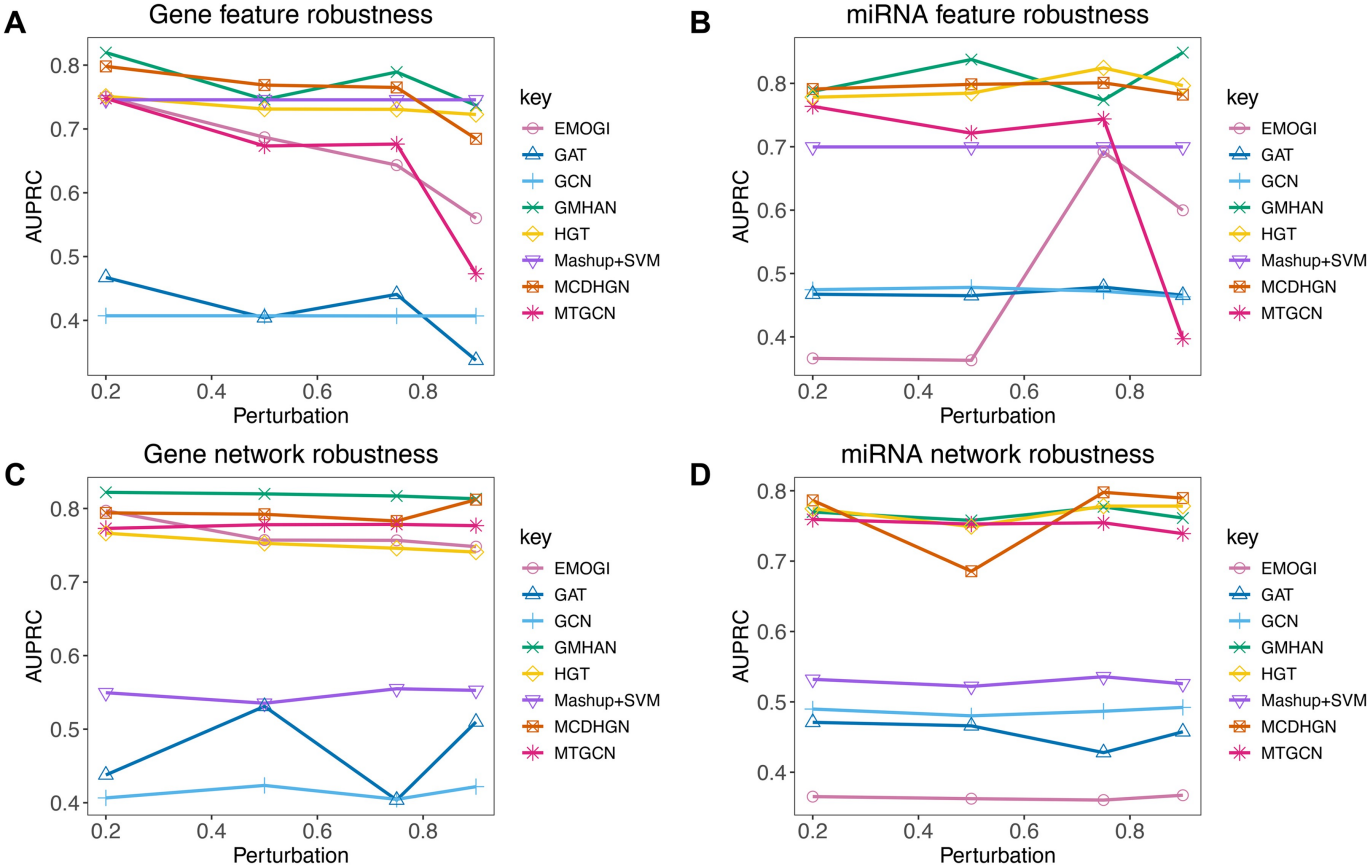
Line plots of AUPR for different methods under various perturbation conditions. (A) AUPR under different perturbation frequencies of gene features. (B) AUPR under different perturbation frequencies of miRNA features. (C) AUPR under different perturbation frequencies of gene networks. (D) AUPR under different perturbation frequencies of miRNA networks. In each panel, the x-axis and y-axis denote perturbation frequency and AUPR, respectively. Different colors and marker shapes indicate various methods.

The robustness of the GMHAN model was evaluated under two perturbation scenarios: feature perturbation and network perturbation. The approach of feature perturbation is to randomly remove features of a certain frequency, while the approach of network perturbation is to randomly remove edges of a certain frequency. The disturbance frequencies were set to 0.2,0.5,0.75 and 0.9. Then, we calculated the AUPR values of different methods at different disturbance frequencies. The results indicated that the AUPR values of the identified coding and non-coding driver genes of GMHAN were the highest at different disturbance frequencies, and the changes were not significant (Fig. 3). The results indicated that GMHAN exhibits robustness under complex interference conditions.

### 3.2 Ablation experiment

An ablation study was conducted on different features, including random features, topological features, individual omics features (gene expression, mutation, and DNA methylation), as well as their pairwise combinations (gene expression + mutation, gene expression + DNA methylation, and DNA methylation + mutation). Model performance was compared based on AUC and AUPR. The results demonstrated that a combination of multi-omics features with topological features yielded the model’s optimal performance (Table 3).

**Table 3:** Ablation experiment results.

Features	Five-fold AUC	Five-fold AUPR
**Mult-omics+node2vec**	**0.902 ± 0.009**	**0.827 ± 0.016**
Mult-omics	0.868 ± 0.006	0.759 ± 0.014
node2vec	0.876 ± 0.015	0.769 ± 0.025
RandomFeaure	0.836 ± 0.028	0.697 ± 0.069
Expr	0.789 ± 0.016	0.607 ± 0.048
Mut	0.818 ± 0.009	0.67 ± 0.026
Meth	0.813 ± 0.005	0.648 ± 0.021
Expr+Mut	0.854 ± 0.007	0.729 ± 0.02
Expr+Meth	0.838 ± 0.009	0.694 ± 0.019
Mut+Meth	0.857 ± 0.005	0.744 ± 0.026

*Note*. node2vec: topological features; Expr: gene expression; Mut: mutation; Meth: DNA methylation. The highest value in each column is highlighted in bold.

### 3.3 Independent dataset performance analysis

**Figure 4:**
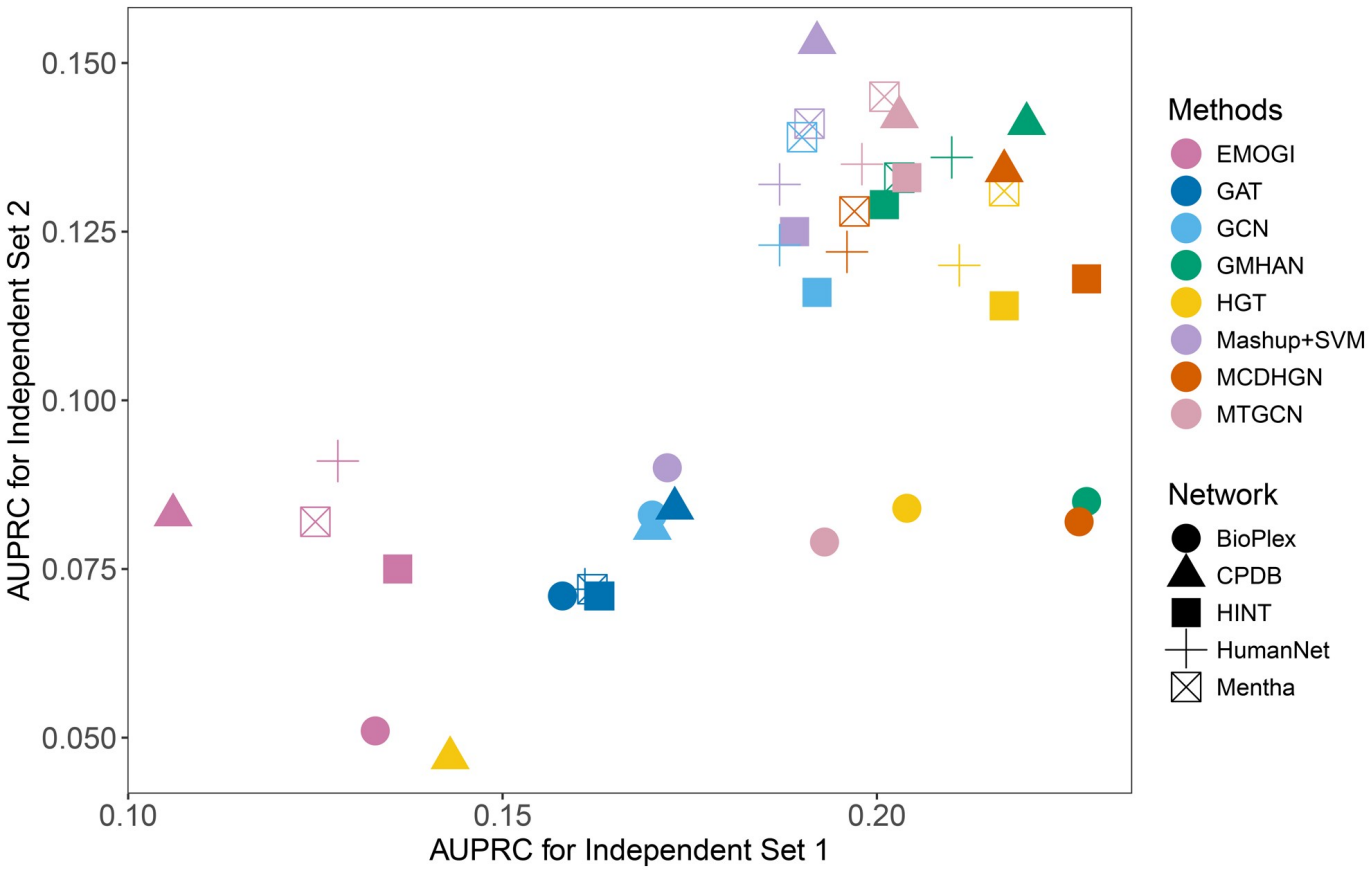
AUPR comparison on independent datasets. The x-axis and y-axis are AUPR on independent set 1 and set 2, respectively. Colors represent methods, and marker shapes represent PPI networks.

To assess whether the constructed model exhibits bias toward specific datasets, we evaluated its performance on two independent datasets. The data in independent dataset 1 were from the OncoKB and ONGene databases. The data in independent dataset 2 were derived from candidate driver genes in the NCG database and genes from Bailey *et al.* (Bailey *et al.* 2018). The construction procedure for the independent datasets is detailed in the Supplementary Material. Notably, the genes in these two datasets do not overlap with the positive sample set used by GMHAN. During evaluation, genes included in the independent datasets were defined as positive samples, while those not included were treated as negative samples. We also compared GMHAN with multiple baseline methods on the two independent datasets. As shown in Fig. 4, GMHAN consistently outperforms all compared methods.

Nevertheless, this sample labeling strategy leads to a reduction in the AUPR. The decline is mainly attributed to the difference in sample labeling between the training set and the independent datasets. In the training set, positive samples are well-validated genes from known cancer driver databases. In the independent datasets, we built the positive set from multiple sources and labeled all other genes as negative. This results in a very small number of positive samples, and some unannotated and hard-to-distinguish potential driver genes may be mixed into the negative set, which together contribute to the drop in AUPR.

### 3.4 Accuracy assessment of driver gene identification in four types of cancer

Next, we applied the GMHAN model to the individual cancer types. BLCA, BRCA, LIHC, and LUAD were selected for in-depth analysis. For each cancer type, the initial gene features comprised methylation profiles, gene expression data, mutation signatures, and 16-dimensional network topological characteristics. The initial miRNA features incorporated miRNA expression levels, differential expression profiles, GIP kernel similarity, and the number of connected genes. Experimental results demonstrated that GMHAN outperformed all comparative methods in predicting both coding and non-coding driver genes in individual cancer types, achieving the highest AUC and AUPR (Table 4).

**Table 4:** Experimental results on different cancer types.

Type of Cancer	Method	Gene		miRNA	
		AUC	AUPR	AUC	AUPR
BRCA	GMHAN	**0.898 ± 0.026**	**0.708 ± 0.033**	**0.887 ± 0.030**	0.740 ± 0.070
	GCN	0.595 ± 0.001	0.13 ± 0.001	0.642 ± 0.003	0.43 ± 0.007
	GAT	0.646 ± 0.001	0.149 ± 0.001	0.671 ± 0.001	0.443 ± 0.001
	HGT	0.878 ± 0.001	0.66 ± 0.003	0.853 ± 0.001	0.783 ± 0.003
	EMOGI	0.304 ± 0.002	0.066 ± 0.001	0.499 ± 0.001	0.281 ± 0.001
	MTGCN	0.740 ± 0.001	0.423 ± 0.001	0.83 ± 0.001	0.681 ± 0.002
	MCDHGN	0.892 ± 0.001	0.687 ± 0.005	0.885 ± 0.001	**0.791 ± 0.005**
BLCA	GMHAN	**0.966 ± 0.019**	**0.798 ± 0.092**	0.891 ± 0.021	0.717 ± 0.079
	GCN	0.469 ± 0.005	0.052 ± 0.001	0.549 ± 0.003	0.28 ± 0.002
	GAT	0.639 ± 0.004	0.093 ± 0.001	0.69 ± 0.008	0.389 ± 0.004
	HGT	0.922 ± 0.002	0.677 ± 0.004	0.871 ± 0.003	0.731 ± 0.021
	EMOGI	0.249 ± 0.004	0.032 ± 0.001	0.511 ± 0.001	0.195 ± 0.001
	MTGCN	0.642 ± 0.001	0.199 ± 0.008	0.858 ± 0.001	0.632 ± 0.003
	MCDHGN	0.946 ± 0.002	0.736 ± 0.007	**0.914 ± 0.001**	**0.785 ± 0.007**
LIHC	GMHAN	**0.909 ± 0.059**	**0.574 ± 0.125**	**0.894 ± 0.021**	**0.771 ± 0.055**
	GCN	0.453 ± 0.001	0.058 ± 0.001	0.605 ± 0.008	0.32 ± 0.001
	GAT	0.615 ± 0.002	0.056 ± 0.001	0.692 ± 0.008	0.411 ± 0.003
	HGT	0.842 ± 0.005	0.469 ± 0.018	0.822 ± 0.003	0.696 ± 0.002
	EMOGI	0.279 ± 0.005	0.027 ± 0.001	0.502 ± 0.001	0.218 ± 0.001
	MTGCN	0.571 ± 0.01	0.094 ± 0.002	0.852 ± 0.001	0.645 ± 0.004
	MCDHGN	0.9 ± 0	0.558 ± 0.002	0.881 ± 0.001	0.742 ± 0.007
LUAD	GMHAN	**0.903 ± 0.059**	0.664 ± 0.128	0.908 ± 0.046	**0.786 ± 0.082**
	GCN	0.599 ± 0.002	0.123 ± 0.001	0.501 ± 0.005	0.261 ± 0.004
	GAT	0.681 ± 0.001	0.163 ± 0.001	0.617 ± 0.011	0.353 ± 0.008
	HGT	0.88 ± 0.001	0.625 ± 0.017	0.844 ± 0.005	0.707 ± 0.017
	EMOGI	0.366 ± 0	0.064 ± 0.001	0.512 ± 0.001	0.216 ± 0.003
	MTGCN	0.641 ± 0.004	0.259 ± 0.008	0.87 ± 0.002	0.646 ± 0.013
	MCDHGN	0.894 ± 0.001	**0.692 ± 0.009**	**0.913 ± 0.001**	0.765 ± 0.01

The highest value in each column is highlighted in bold.

### 3.5 Identification and analysis of novel pan-cancer driver genes

**Figure 5:**
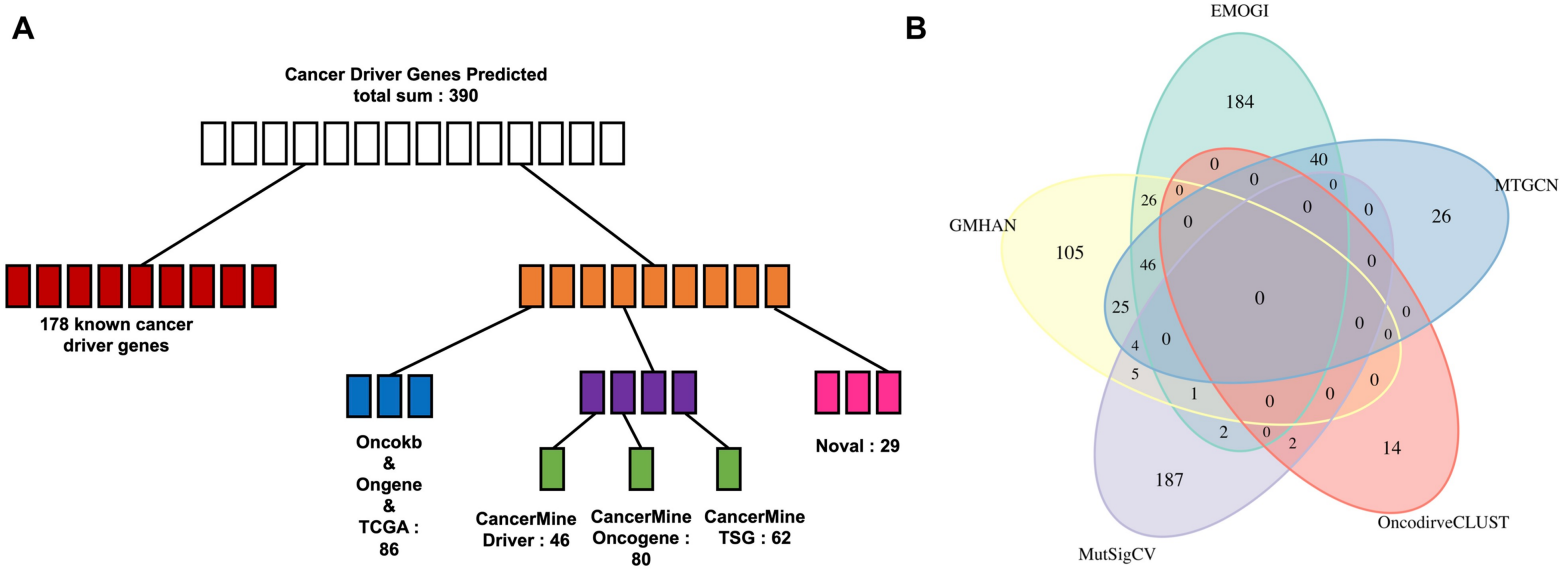
Results of novel driver gene prediction from GMHAN model. (A) Hierarchical graph of novel driver gene prediction from GMHAN. (B) Venn diagram comparing GMHAN with other methods in predicting novel cancer driver genes.

The model was trained using all data to predict probability scores for all genes. Genes with a prediction probability greater than 0.8 were defined as driver genes, yielding a total of 390 candidate genes. By taking the intersection between these candidate genes and the known driver gene set (positive samples), 178 known driver genes were identified. For the remaining 212 novel predicted genes, further functional validation analysis was conducted. Among the genes predicted by GMHAN, 86 were validated against in OncoKB (Chakravarty *et al.* 2017) database, the ONGene (Liu *et al.* 2017) database, and the pan-cancer driver gene list (Ding *et al.* 2018). And most of the remainder had literature support in CancerMine (Lever *et al.* 2019) database as driver or cancer-associated genes (Fig. 5A). In summary, 92.56% novel driver genes predicted by the GMHAN model were supported by at least one independent source. Subsequently, we compared GMHAN’s predictions of novel driver genes against the results of EMOGI, MutSigCV, MTGCN, and OncodriverCLUST (Fig. 5B). Our analysis showed that about half of the new genes identified by GMHAN have not been reported by any other methods.

Next, we used ATAD2 and DLX4 as examples to prove the GMHAN’s performance in predicting novel driver genes. ATAD2, a highly conserved bromodomain-containing protein (Liu *et al.* 2022) is frequently overexpressed in gastrointestinal, lung, and urological cancers (Zheng *et al.* 2015), and plays a significant role in promoting tumorigenesis. Its oncogenic function is orchestrated through multiple mechanisms. Primarily, ATAD2 modulates the Rb-E2F1 signaling pathway to control cell cycle progression. It further cooperates with transcription factors to upregulate proliferative and anti-apoptotic genes (Li *et al.* 2024). Additional studies implicate ATAD2 in the PI3KAKT and TGF-β1SMAD3. In colorectal cancer, ATAD2 directly interacts with the EMT factor WTIST1 to enhance MYC transcription, a mechanism that promotes malignancy (Roy 2024). This multifaceted participation in the key cancer pathway strongly supports its potential as a driver gene.

DLX4, a Distal-less homeobox (DLX) family gene, is frequently upregulated in cancers where it contributes to early tumorigenesis and immune regulation. In ovarian cancer, DLX4 stimulates IL-1βNF-κB signaling to induce CD44, enhancing tumor-mesothelial interactions and peritoneal metastasis (Wang *et al.* 2023). In addition, it can act as a key disruptor of the TGF-β pathway, effectively neutralizing a major obstacle to tumor suppression by interfering with the tumor suppressor signaling axis (Xia *et al.* 2015). Studies demonstrated that DLX4 interacts with SMAD4, thereby preventing the formation of functional SMAD234 transcriptional complexes. Consequently, this interaction disrupts the transcription TGF-β-mediated cell cycle inhibitors p15Ink4B and p21WAF1/Cip1, thereby counteracting TGF-β’s anti-proliferative property (Trinh 2011).

### 3.6 Enrichment analysis of the novel driver genes predicted by GMHAN

**Figure 6:**
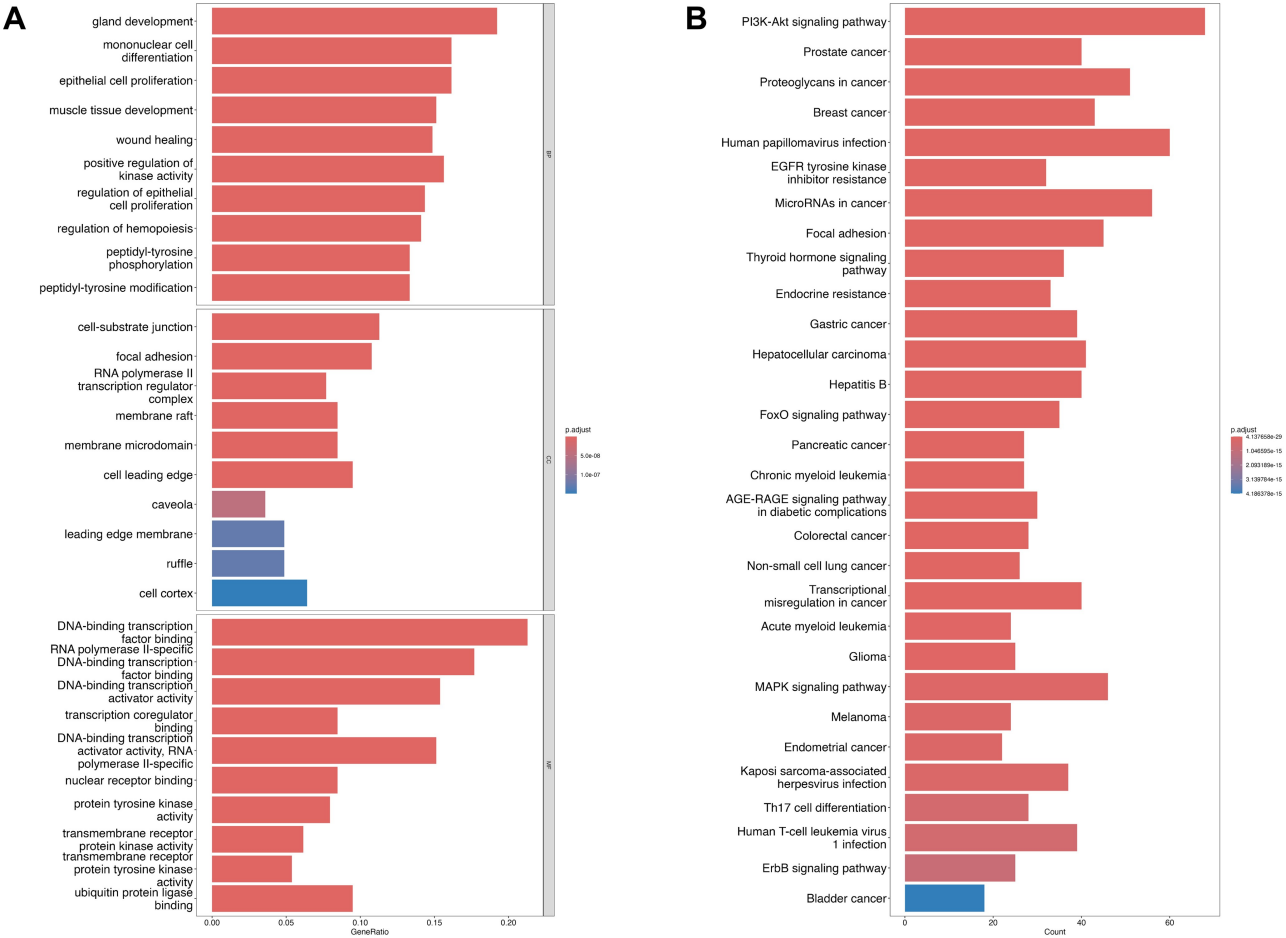
Bar plots of GO and KEGG enrichment analyses for driver genes predicted by GMHAN.

Enrichment analysis were conducted on GMHAN predicted driver genes. GO enrichment analysis showed that multiple GO terms related to cancer development were significantly enriched (Fig. 6A). For instance, the term “epithelial cell proliferation” is significantly enriched, and epithelial cell proliferation is an important indicator for assessing the risk of adenocarcinoma. The results of KEGG enrichment analysis indicated that 137 KEGG pathways were significantly enriched. We selected the 30 most significantly enriched pathways to draw a bar chart (Fig. 6B). These include multiple pathways related to “Breast cancer,” “Prostate cancer,” and “Gastric cancer.” Notably, the “PI3K-Akt signaling pathway” was significantly enriched. Evidence suggests that the excessive activation of PI3K-Akt signaling promotes tumor proliferation and drug resistance in various cancers (Khezri *et al.* 2022).

## 4 Discussion

Accurately identifying driver genes is essential for providing precise treatment to cancer patients. Thanks to the rapid accumulation of sequencing data, many driver genes identification methods have been developed. However, non-coding genes are also important in cancer development. Therefore, we proposed the GMHAN model that leverages heterogeneous graph attention to identify both coding and non-coding cancer driver genes. This model was constructed by first integrating a heterogeneous network comprising genes and miRNAs, and incorporating multi-dimensional features including multi-omics data; topological information from gene interaction networks; and diverse molecular features of miRNAs. Building on this framework, GMHAN employed a heterogeneous graph attention mechanism to achieve synergistic identification of coding and non-coding driver genes. Our model improves the accuracy of driver gene identification and effectively extends the scope of detection to non-coding genes.

Driver genes are important to cancer development and progression. They already have relatively complete functional annotations and clear interaction patterns. By introducing multi-source features and the heterogeneous network, GMHAN still achieved good performance in coding gene prediction, although the performance gain was relatively small. Future efforts will aim to boost coding driver gene prediction accuracy by refining the model structure.

For the prediction of non-coding driver miRNAs, available functional annotations are relatively limited, and their regulatory roles are mostly reflected in indirect associations across different molecules and multiple biological pathways. By integrating heterogeneous relationships of miRNA-gene and miRNA-miRNA, GMHAN exhibits obvious advantages in aggregating multi-path semantic information and characterizing complex biological interactions. Accordingly, our model achieves a more prominent performance improvement in the prediction task of non-coding driver miRNAs.

In this study, GMHAN identified 212 novel driver genes. Of these, 92.56% were found in at least one cancer database, and most had not been reported by other methods. GO and KEGG analysis further showed significant enrichment in cancer-related pathways. These results suggest that these genes have potential biological relevance. They may serve as new targets for understanding the molecular mechanisms of tumor development or as early diagnostic markers, and their functions could be further validated through cell and animal experiments.

Although GMHAN has achieved promising results, there is still room for improvement. First, when integrating coding gene features, GMHAN simply concatenates multi omics features and topological features. In the future, we will explore more complex integration strategies to capture cross modality complementary information. Second, the current model does not fully make use of edge weights in the heterogeneous network. Subsequent work will consider introducing a weighting mechanism to better characterize the strength of associations between nodes. Third, we built the miRNA-miRNA network using Pearson correlation of miRNA expression. The main reasons are this method is computationally efficient and easy to interpret, and well suited for large-scale co-expression network analysis. It has been widely used in existing studies (Chaudhary *et al.* 2021; Yu and Yu, 2022). Moreover, prior knowledge of miRNA-miRNA regulatory relationships is currently limited. Pearson correlation allows construction without relying on any prior regulatory information, and it also achieved satisfactory performance in our proposed GMHAN model. For the above reasons, we adopted Pearson correlation to build the miRNA-miRNA network. Nevertheless, this method has some limitations. It only captures linear correlation and is sensitive to outliers in expression data. In future work, we will explore alternative network construction strategies, such as mutual information algorithms or building networks based on miRNAs sequence similarity. Fourth, the completeness of multi omics data also affects model performance. For cancer types with missing multi omics data, the generalizability of the model still needs further evaluation and validation.

## 5 Conclusion

We built GMHAN to identify coding and non-coding driver genes. It integrates multi-omics data with gene-miRNA network, the model adopts HAN to learn deep representations of gene and miRNA simultaneously. On this basis, the scope of driver gene identification is extended from coding genes to non-coding genes. Evaluated on pan-cancer and cancer-specific datasets, GMHAN achieves higher AUC and AUPR than baseline methods, which demonstrates its effectiveness and robustness in identifying coding and non-coding driver genes. Although the GMHAN model still has room for improvement, it nevertheless serves as a powerful tool for systematic cancer driver gene analysis.

## Supplementary Material

btag403_Supplementary_Data

## Data Availability

The data used in this study are publicly available from TCGA at https://portal.gdc.cancer.gov/. The source code of GMHAN is available at: https://github.com/mping315/GMHAN and https://doi.org/10.5281/zenodo.20154736.
