# Supplementary material for: GMHAN: a heterogeneous graph attention framework for prioritizing coding and non-coding driver genes
Source: Bioinformatics. 2026 Jun 18;42(6):btag403. doi: 10.1093/bioinformatics/btag403 (PMC13310458; doi:10.1093/bioinformatics/btag403)
Supplement: btag403_Supplementary_Data [file btag403_supplementary_data.docx]

# Materials and methods

## The biological features of genes:

These biological characteristics comprise three key metrics. For each cancer type, the calculation methods for the frequency of gene mutations is the ratio of SNVs to the length of their exon regions. We calculate the differential DNA methylation rate by computing the mean absolute difference of methylation signals between all cancerous and healthy tissue samples, formulated as:

|  | ${dm}_{i}^{c}=\frac{1}{\left\vert S_{c} \right\vert}\sum_{s \in S_{c}} \left( \beta_{i}^{t}-\beta_{i}^{n} \right)$ | () |
| --- | --- | --- |

where ${dm}_{i}^{c}$ represents the methylation signal difference of gene i from cancer c; $\beta_{i}^{t}$ represents methylation signal value in cancer samples; $\beta_{i}^{n}$ represents the methylation signal value in normal samples and S represents the samples.

Similarly, the gene differential expression rate corresponds to the mean log2-fold change of expression levels between tumor samples and matched normal controls. Finally, we performed horizontal concatenation of the three types of biological features and applied Min-Max normalization.

## The benchmark methods in this study include:

**GCN**

GCN (Kipf, 2016), a deep learning method for graph-structured data. This study used a three-layer GCN for node classification. The dimensions of the hidden layer are 200 and 100. Dropout is 0.25. Adam optimizer with learning rate of 0.001, weight decay is 0.001.

**GAT**

GAT (Veličković, et al., 2017) is a graph neural network model that introduces an attention mechaism. This study used a three-layer GAT for node classification. The dimensions of the hidden layer are 200 and 100. Dropout is 0.25. Adam optimizer and learning rate of 0.001, weight decay is 0.001.

**EMOGI**

EMOGI (Schulte-Sasse, et al., 2021) is an interpretable deep learning framework for predicting and interpreting pan-cancer driver genes. The specific GCN architecture features two hidden layers with dimensions of 300 and 100. Dropout is 0.5. Adam optimizer and learning rate of 0.001, weight decay is 0.005.

**HGT**

HGT (Heterogeneous Graph Transformer)(Hu, et al., 2020) is specifically designed for heterogeneous graphs. The model employs a two-layer HGT architecture and is equipped with 4 attention heads. AdamW optimizer and learning rate is 0.001.

**MCDHGN**

MCDHGN (Wang, et al., 2024) is a heterogeneous graph node classification model extended from HAN. The model has a hidden layer dimension of 256, 4 attention heads, dropout rate is 0.4, and earning rate is 0.0001.

# The two independent datasets included in this study

**Independent Set 1**

Comprises cancer genes sourced from the OncoKB (Chakravarty, et al., 2017) database, selected based on the clinical actionability of their alterations and their pivotal roles in cancer biology, along with cancer genes from the ONGene (Liu, et al., 2017) database, which employs an integrated approach combining large-scale cancer genomic data with mutation frequency-based statistical methods and functional impact prediction algorithms to systematically identify cancer driver genes.

The OncoKB database mainly includes genes that, after mutation, can affect the development of cancer. The ONGene database focuses on some new and potentially influential cancer genes.

**Independent Set 2**

Includes candidate cancer genes from the NCG (Repana, et al., 2019) database, as well as high-confidence driver genes identified by Bailey et al (Bailey, et al., 2018). We first removed the overlapping genes between the independent dataset and the positive/negative sample sets. The deduplicated independent dataset was then explicitly defined as positive samples, while the remaining genes were designated as negative samples.

# References

Bailey, M.H.*, et al.* Comprehensive characterization of cancer driver genes and mutations. *Cell* 2018;173(2):371-385. e318.

Chakravarty, D.*, et al.* OncoKB: a precision oncology knowledge base. *JCO precision oncology* 2017;1:1-16.

Hu, Z.*, et al.* Heterogeneous graph transformer. In, *Proceedings of the web conference 2020*. 2020. p. 2704-2710.

Kipf, T. Semi-supervised classification with graph convolutional networks. *arXiv preprint arXiv:1609.02907* 2016.

Liu, Y., Sun, J. and Zhao, M. ONGene: A literature-based database for human oncogenes. *Journal of genetics and genomics* 2017;44(2):119-121.

Repana, D.*, et al.* The Network of Cancer Genes (NCG): a comprehensive catalogue of known and candidate cancer genes from cancer sequencing screens. *Genome biology* 2019;20(1):1.

Schulte-Sasse, R.*, et al.* Integration of multiomics data with graph convolutional networks to identify new cancer genes and their associated molecular mechanisms. *Nature Machine Intelligence* 2021;3(6):513-526.

Veličković, P.*, et al.* Graph attention networks. *arXiv preprint arXiv:1710.10903* 2017.

Wang, L.*, et al.* MCDHGN: heterogeneous network-based cancer driver gene prediction and interpretability analysis. *Bioinformatics* 2024;40(6):btae362.
